# Novo molde impresso em três dimensões para substituição do calcâneo após calcanectomia total por um tumor de células gigantes agressivo recorrente do calcâneo

**DOI:** 10.1055/s-0045-1809518

**Published:** 2025-07-01

**Authors:** Goh Boay Heong Eyrique, Tee Kok Keat, Aaron Gerarde Paul

**Affiliations:** 1 Unidade de Oncologia Ortopédica, Ortopedia e Traumatologia, Hospital Queen Elizabeth I and II Sabah, Kuala Lumpur, Malásia

**Keywords:** calcâneo, impressão tridimensional, oncologia, tumores de células gigantes, calcaneum, giant cell tumors, oncology, printing, three-dimensional

## Abstract

Avanços no processamento de imagens levaram ao uso clínico da tecnologia de impressão tridimensional (3D), dando aos cirurgiões modelos físicos anatômicos realistas para recriação precisa de estruturas ósseas. Moldes impressos em 3D podem desempenhar um papel essencial em substituições cirúrgicas, oferecendo eficiência e boa relação custo-benefício. Este relato de caso descreve a criação inovadora de um calcâneo anatômico usando uma impressora 3D. Um paciente apresentou uma recidiva de tumor de células gigantes do calcâneo em estágio agressivo, com necessidade de ressecção completa, deixando um grande espaço vazio que exigia reconstrução. Este artigo descreve a metodologia utilizada desde a impressão 3D pré-operatória do protótipo, passando pelo procedimento cirúrgico, até os cuidados pós-operatórios. As imagens 3D do calcâneo foram extraídas de uma tomografia computadorizada e editadas usando um *software* de modelagem 3D para impressão do osso como uma concha oca. O protótipo impresso foi criado em duas metades e enviado para esterilização a gás. Após a ressecção do calcâneo acometido, o molde foi preenchido com cimento ósseo e as duas metades foram unidas, com uma tela de prolene entre elas, para fixação nos tecidos moles. Após a cura do cimento ósseo, a concha foi removida e o calcâneo moldado foi implantado no paciente com parafusos. Os tecidos moles circundantes e o tendão calcâneo foram suturados à tela. No pós-operatório, o paciente utilizou uma placa dorsal de gesso por 6 semanas para permitir a incorporação dos tecidos moles. Seis semanas após a cirurgia, o paciente iniciou atividades com suporte de peso no pé esteticamente moldado. Este método de reconstrução óssea é eficiente, econômico e reprodutível.

## Abstract (en)

Advances in image processing have led to the clinical use of 3D printing technology, providing surgeons with realistic physical models of anatomy that enable them to recreate accurate bone structures. 3D-printed molds can play a central role in surgical replacements, offering both efficiency and cost-effectiveness. This case report describes the innovative creation of an anatomical calcaneum using a 3D printer. A patient presented with a recurrent aggressive stage of calcaneal giant cell tumor, which necessitated a complete resection, leaving a large void that required reconstruction. This paper outlines the 3D printing methodology used from pre-operative printing of the prototype, through the surgical procedure, to post-operative care. 3D images of the calcaneum were extracted from a CT scan and edited using 3D modelling software to print a hollow-shelled calcaneum. The printed prototype was created in two halves and sent for gas sterilization. After resecting the diseased calcaneum, the mold was filled with bone cement and clasped together with a proline mesh in between, for soft tissue attachment. Once the cement set, the shell was removed, and the shaped bone cement calcaneum was implanted into the patient with screws. The surrounding soft tissues and Achilles tendon were sutured to the mesh. Post-operatively, the patient was kept in a plaster dorsal slab for six weeks to allow for soft tissue incorporation. Six weeks after surgery, the patient began weight-bearing activities with an aesthetically shaped foot. This method for bone reconstruction is efficient, economical, and reproducible.

## Introdução

Os avanços no processamento de imagens levaram ao uso clínico crescente da tecnologia de impressão tridimensional (3D). As imagens de tomografia computadorizada (TC) ou ressonância magnética (RM) podem ser convertidas em arquivos 3D e gerar um modelo anatômico perfeito. Essa tecnologia confere versatilidade ao processo de *design* e possibilita a produção eficiente de anatomia personalizada e pronta para uso, adaptando-se às especificidades necessárias. 1 Na cirurgia ortopédica oncológica, moldes e instrumentos impressos em 3D podem ser utilizados para restauração óssea após uma ressecção tumoral ampla. Existem opções convencionais de substituição com implantes metálicos, mas seus custos podem ser exorbitantes. Em nosso caso, a concha anatômica em 3D foi impressa a um custo mínimo e o cimento ósseo utilizado para preenchê-la também teve baixo custo. Este artigo analisa a técnica cirúrgica, descreve os fundamentos da tecnologia de impressão 3D e discute suas possíveis aplicações em cirurgia ortopédica e impacto futuro.

## Relato de Caso

**Fig. 1:**
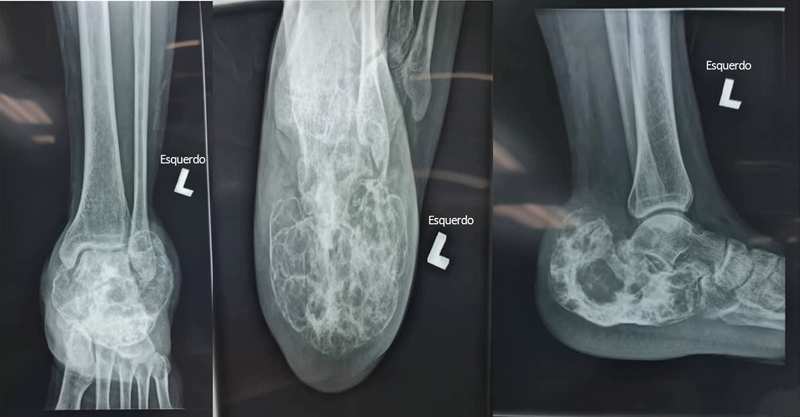
Imagem radiográfica do calcâneo esquerdo acometido em ( *A* ) vistas anteroposterior, ( **B** ) axial e ( **C** ) em perfil.

Este tratamento cirúrgico inovador foi realizado em um paciente que chegou à nossa instituição com um tumor de células gigantes (TCG) recorrente do calcâneo. O paciente havia sido tratado 18 meses antes com uma série de denosumabe e curetagem extensa e apresentava aumento de volume e dor no calcanhar esquerdo ( Figs. 1 e 2 ).

**Fig. 2:**
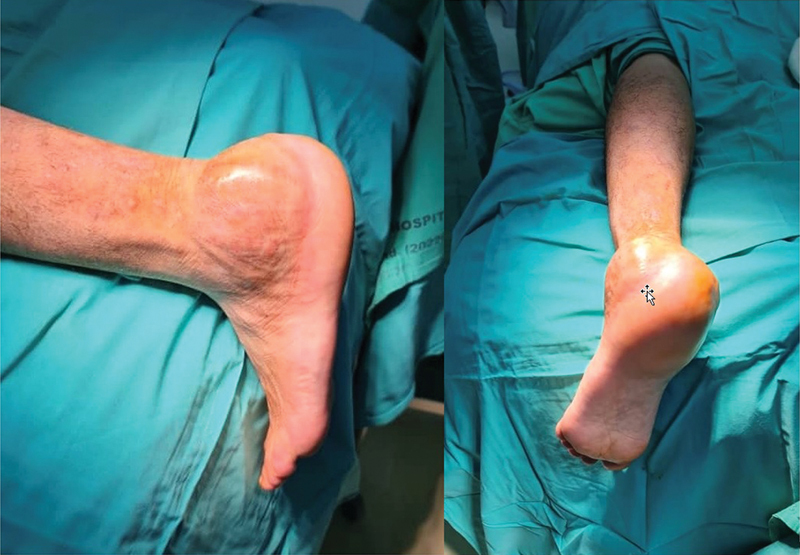
Quadro clínico do pé esquerdo acometido.

## Descrição da Técnica Impressão 3D

- Planejamento pré-operatório

Há diversos métodos de impressão 3D, mas todos compartilham os mesmos princípios e processos. 1 Embora a impressão 3D tenha ganho força ao longo dos anos, não é amplamente difundida devido à sua íngreme curva de aprendizado e conhecimento limitado. 2 Como este paciente apresentava um tumor, era crucial manter os altos padrões dos princípios de ressecção oncológica. O paciente passou pela avaliação clínica padrão, com exames de imagem (TC e RM) do pé para delinear a extensão de acometimento dos tecidos moles e neurovascular. Uma vez estabelecido o diagnóstico, as margens de ressecção cirúrgica foram determinadas e previram uma cavidade significativa, já que uma calcanectomia total foi planejada. O calcâneo removido precisava ser substituído ou reconstruído para facilitar a sustentação de peso. Uma TC do calcâneo contralateral normal foi utilizada para a criação do molde.

- Materiais necessários para a cirurgia

**Fig. 3:**
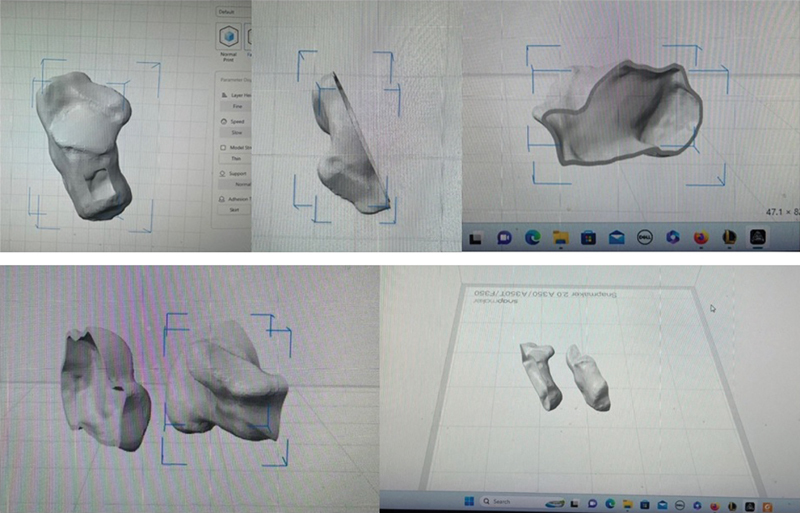
( **A** ) Criação do modelo tridimensional (3D) a partir da reconstrução 3D da tomografia computadorizada. ( **B** ) Vista superior da metade criada do calcâneo. ( **C** ) Vista sagital da metade criada do calcâneo. ( **D** ) Criação de uma imagem espelhada para impressão. ( **E** ) *Designs* finais para impressão 3D.

A cirurgia exigiu uma impressora 3D padrão, como, por exemplo, Kokoni Smart, Bambulab e Creality. Além disso, houve necessidade de equipamentos de TC e RM para obtenção dos modelos 3D, gerados em Slicer (slicer.org). Os modelos foram importados para um *software* de modelagem 3D (Fusion 360) ( Fig. 3 ). Os *designs* finais poderiam ter sido impressos com utilização de diversos programas específicos. O modelo da impressão anatômica foi preparado antes da cirurgia e esterilizado com gás, pois seu material, ácido polilático (PLA), era termolábil.

- Indicações e contraindicações

Este método de substituição é empregado após uma excisão local ampla do tumor subjacente, evitando a necessidade de amputação. A cirurgia de salvamento de membro compreende técnicas cirúrgicas para ressecção de tumores musculoesqueléticos de extremidades, seguida pela reconstrução do membro acometido de forma funcional e cosmeticamente aceitável. 3 As contraindicações relativas são fraturas patológicas, encapsulamento neurovascular e mau posicionamento do trato de biópsia. A cirurgia de salvamento de membro é o tratamento preferencial para tumores musculoesqueléticos de extremidades na era moderna, pois demonstrou não comprometer a sobrevida ou a recidiva em comparação à amputação. 3

- Anatomia cirúrgica

**Fig. 4:**
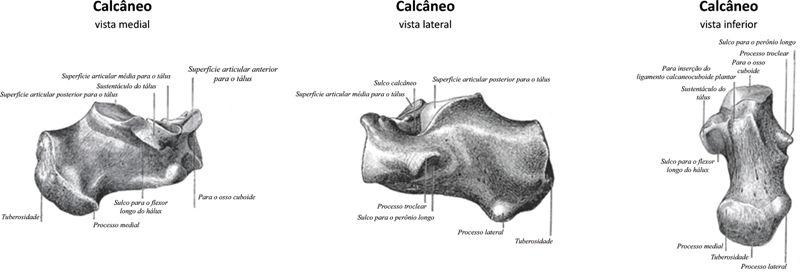
Anatomia do calcâneo. ( **A** ) Medial, ( **B** ) lateral e ( **C** ) inferior. Fonte: Luijkx T, Elthokapy M, Gregory L, et al. Calcaneus. Reference article, Radiopaedia.org.

O calcâneo é um osso irregular, de formato aproximadamente cúbico, situado abaixo do tálus, e forma o núcleo do calcanhar ( Fig. 4 ). 4 A parte posterior do calcâneo é circular e tem três facetas. A faceta superior é separada do tendão calcâneo pela bursa retrocalcânea. A faceta média é o sítio de inserção do tendão do calcâneo. A faceta inferior é contínua à tuberosidade calcânea na superfície plantar. Superiormente, há uma faceta revestida de cartilagem (faceta articular talar média) direcionada à faceta média correspondente da cabeça do tálus como parte da articulação subtalar. A superfície anterior possui uma faceta articular convexa para a articulação do osso cuboide.

- Técnica cirúrgica

**Fig. 5:**
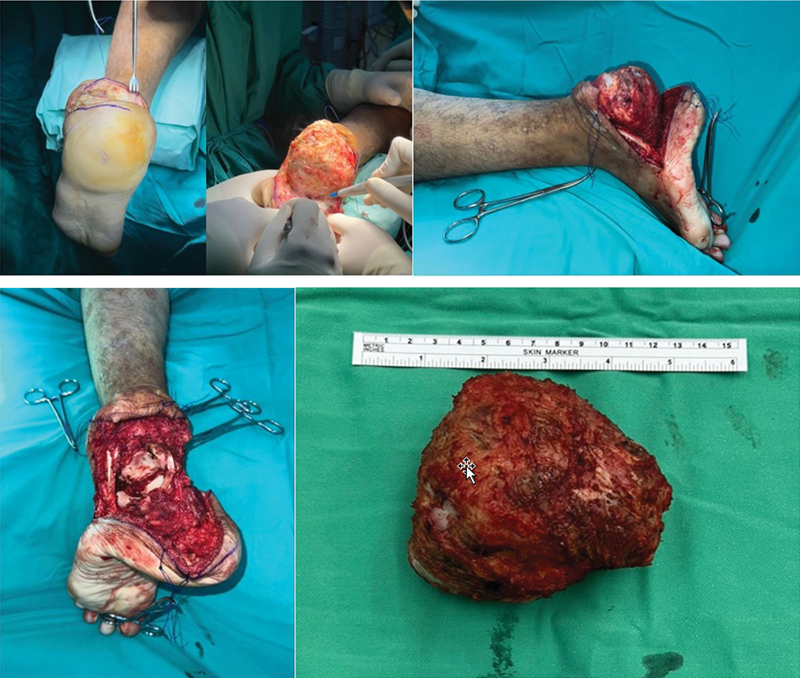
Calcanectomia total com abordagem de Cincincati. ( **A** ) Incisão da abordagem de Cincincati sobre o calcanhar. ( **B** ) Ressecção ampla do calcâneo acometido. ( **C** ) Vista sagital do calcâneo acometido. ( **D** ) Espaço vazio após a calcanectomia total. ( **E** ) Tumor de células gigantes recorrente excisado do calcâneo.

Todos os pacientes devem ser submetidos à ressecção oncológica com princípios rigorosos, ou seja, a ressecção em bloco com margem livre. A ressecção cirúrgica é crucial no tratamento de tumores sólidos primários. A ressecção das margens do tumor ainda é preocupante, já que sua realização inadequada facilita a recidiva da doença. 5 Após a excisão do tumor, é importante assegurar que não haja retenção macroscópica da neoplasia. Em nosso relato de caso, a cirurgia foi realizada em decúbito ventral, utilizando a incisão de Cincinnati 6 ( Fig. 5 ). O procedimento foi uma calcanectomia total com ressecção em bloco ( Fig. 5 ). O tríceps sural foi marcado para reconstrução posterior.

**Fig. 6:**
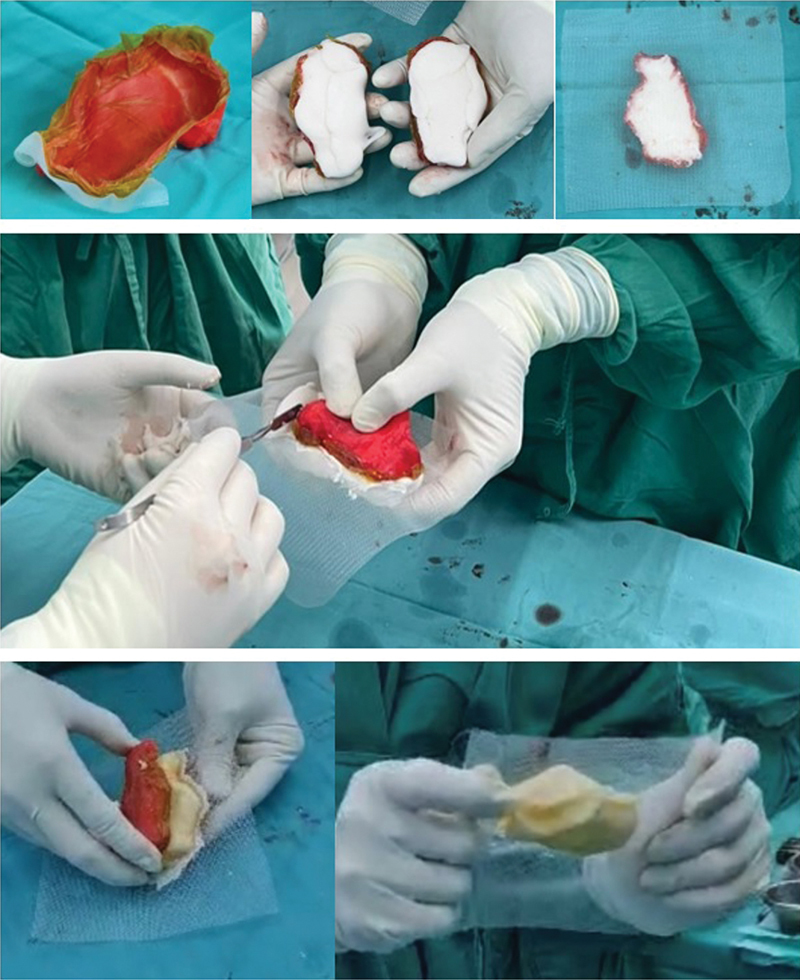
Moldagem do protótipo de modelo do calcâneo com cimento ósseo. ( **A** ) Revestimento do molde com Ioban e parafina líquida. ( **B** ) As duas metades do molde são preenchidas com um pacote de cimento ósseo padrão. ( **C** ) Uma bainha de tela de prolene é colocada entre as metades antes da sua união para posterior consolidação. ( **D** ) Fixação das metades do molde com cimento e tela de prolene enquanto o assistente remove o cimento extrudado. ( **E** ) Retirada do molde impresso do calcâneo de cimento. ( **F** ) O calcâneo de cimento com a tela de prolene incorporada no centro para fixação em tecido mole.

O molde impresso esterilizado do calcâneo foi preparado para implantação. Cada metade do molde impresso foi revestida com campo cirúrgico Ioban e parafina líquida ( Fig. 6 ). Em seguida, cada metade do molde foi preenchida com um pacote de cimento ósseo padrão. As duas metades foram fixadas, com uma tela de prolene entre elas. A tela de prolene atua como âncora para a reconstrução do tecido mole. O cimento extrudado foi removido ainda na forma líquida, antes do endurecimento ( Fig. 6 ).

**Fig. 7:**
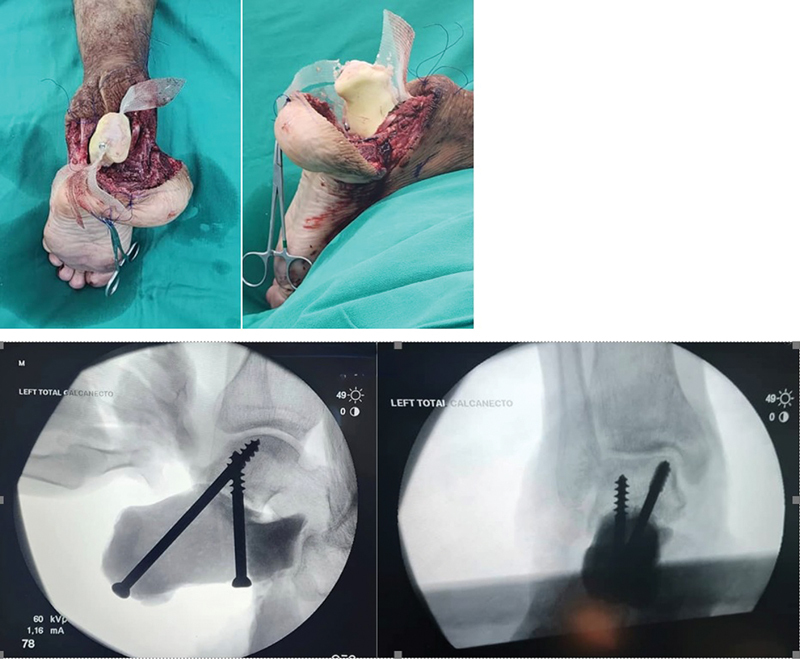
O calcâneo de cimento inserido no espaço ressecado para reconstrução. ( **A** ) Vista posterior. ( **B** ) Vista lateral. Vistas intraoperatórias do intensificador de imagem dos dois parafusos esponjosos inseridos. ( **C** ) Vista lateral. ( **D** ) Vista anteroposterior.

Devido às propriedades físicas do PLA, a casca endurecida do molde amolece durante a fase exotérmica da cura do cimento, permitindo sua fácil remoção ( Fig. 6 ). O calcâneo moldado foi inserido no espaço ressecado. O tríceps sural foi suturado à face posterior do calcâneo na tela de prolene, que foi aparada nas áreas de inserção. A articulação talocalcânea foi estabilizada com a inserção de parafusos esponjosos com rosca parcial ( Fig. 7 ). Brocas de tamanhos crescentes foram usadas para dilatação gradual do trajeto, evitando rachaduras no cimento.

Após a inserção dos parafusos, o tendão calcâneo foi suturado à tela sobre a parte posterior do calcâneo com fio Ethibond tamanho 2. A articulação calcaneocuboide foi reconstruída com a tela ao redor dessa articulação e do ligamento circundante. Alternativamente, outro parafuso poderia ser inserido em sentido posterior, cruzando a articulação calcaneocuboide ( Fig. 7 ).

Um dreno foi colocado e o fechamento do tecido subcutâneo e da pele foi feito com Vicryl 1 e Dafilon 3/0, respectivamente. A ferida foi coberta com creme antibiótico e gesso, complementado com gaze macia e curativo.

- Manejo pós-operatório

O dreno foi mantido por 3 a 5 dias até a epitelização da ferida. As suturas foram removidas 2 semanas após a cirurgia. Uma placa dorsal abaixo do joelho foi mantida por 8 semanas depois do procedimento. Posteriormente, houve movimentação passiva e ativa progressiva do tornozelo sem peso por mais 6 semanas para permitir a incorporação do tendão calcâneo. A sustentação parcial do peso com muletas foi permitida 6 semanas após a cirurgia e a sustentação total foi permitida aos 3 meses. Este paciente foi acompanhado com exames clínicos e radiológicos periódicos. Os resultados funcionais foram avaliados de acordo com o sistema proposto pela *International Society of Limb Salvage* e aprovado pela *Musculoskeletal Tumor Society* . 7

- Resultado e Acompanhamento

**Fig. 8:**
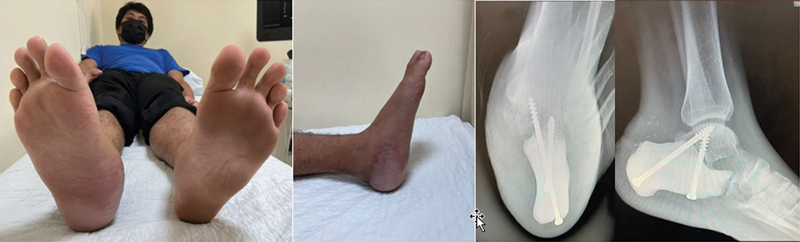
Imagem clínica de ( **A** ) pé bilateral em vista plantar e ( **B** ) lateral. Radiografia ( **C** ) axial do calcâneo e ( **D** ) em perfil do pé aos 9 meses de acompanhamento após a cirurgia.

O paciente foi acompanhado às 2 e 6 semanas e 3 e 6 meses após a cirurgia. A cicatrização foi satisfatória, sem deiscência ou necrose do coxim adiposo, e o paciente iniciou a deambulação completa após 3 meses. O paciente conseguia caminhar com marcha razoavelmente equilibrada e a radiografia mostrou a integridade do implante impresso e dos parafusos impressos ( Fig. 8 ). O paciente ficou muito satisfeito com o resultado e a reabilitação.

- Pérolas (dicas e truques) e desafios
  ○ Tamanho e morfologia
    ▪ Ajuste anatomicamente confortável sem interferência nas estruturas circundantes
    ▪ Tamanho correto
    ▪ A impressão de uma casca de 2 mm de espessura é suficiente para assegurar a resistência e a maleabilidade durante o endurecimento exotérmico do cimento.
  ○ Moldagem
▪ É importante medir a quantidade necessária de cimento para preenchimento do espaço sem porosidade interna. Esta quantidade de cimento é estimada a partir do volume da estrutura, que pode ser calculado com o *software* de *design* 3D.
    ▪ Pressão constante e precisão na manutenção do fechamento durante o endurecimento do cimento
  ○ Resistência mecânica
    ▪ Manutenção da integridade do implante com suporte de carga
    ▪ Fixação estável às estruturas adjacentes
  ○ Fixação
    ▪ Perfuração com brocas de tamanho crescente para evitar rachaduras no cimento
    ▪ Uso de parafusos com rosca parcial na articulação para permitir seu microdeslizamento durante a deambulação
    ▪ Evitar muitas fixações ao redor do cimento, uma vez que causam rigidez excessiva
    ▪ Há necessidade de determinação prévia da qualidade, do comprimento, do tamanho, do tipo e dos tratos
    ▪ Considerar fixações adicionais para manutenção da boa estabilidade
  ○ Reconstrução do tecido mole
    ▪ Fixação estável e forte dos tecidos moles, por exemplo, tendão calcâneo e cápsula sobre a articulação calcaneocuboide.
- Complicações
  ○ Infecção profunda e superficial
  ○ Recidiva em caso de ressecção inadequada das margens do tumor
  ○ Necrose do retalho cutâneo
  ○ Fratura do implante com sustentação de cargas elevadas
  ○ Rigidez articular levando ao desenvolvimento precoce de osteoartrite secundária
  ○ Falência do implante (fadiga do parafuso)
- Singularidade da técnica dos autores em comparação à técnica padrão

Este método de impressão 3D indireta pode ser reproduzido e seu custo é significativamente menor do que o da impressão 3D direta em metal. O custo de impressão do molde é mínimo. A maior parte do custo se refere ao cimento ósseo utilizado. Este método permite o ajuste anatômico com distribuição de força quase fisiológica durante a sustentação da carga, diferentemente de um espaçador de cimento com formato irregular.

## Discussão

A recidiva de um tumor previamente tratado, seja maligno ou benigno agressivo, exige ressecção mais ampla e extensa, deixando um vazio significativo. Esses vazios precisam ser preenchidos para permitir a distribuição de forças para suporte de carga. A reconstrução requer uma análise pré-operatória 3D completa da deformidade ou substituição estrutural para restauro do alinhamento do retropé e deambulação com sustentação total de peso. Esse vazio complexo apresenta desafios significativos, daí a opção de utilizar a tecnologia de impressão 3D na cirurgia de reconstrução. Como a substituição do calcâneo ressecado é secundária, o fator mais importante ainda é manter a ressecção com margem livre do tumor recorrente. A impressão 3D médica foi introduzida pela primeira vez para avaliação de fratura intra-articular do calcâneo em 1997 por Kacl et al. 8 Apesar do lapso de muitos anos e da redução do custo de produção, o custo total da impressão de todo o calcâneo em titânio ainda representa um fardo financeiro importante. 9 A impressão 3D evoluiu ao longo dos anos, permitindo o planejamento pré-operatório, placas de pré-contorno, placas de pré-moldagem e a fabricação de guias específicos para o paciente, entre outros. 9 Devido à complexidade do pé e do tornozelo, esse método definitivamente ajudou o estabelecimento de uma fixação estável e a substituição anatômica sem custo econômico significativo.

## Considerações Finais

Esta abordagem inovadora não só promete recuperação funcional, como também enfatiza o conforto do paciente devido ao menor tempo de recuperação e melhor ajuste anatômico. Trata-se de um método eficiente, econômico e reprodutível para reconstrução óssea após a ressecção completa ou parcial de tumores ósseos. A evidência de um único caso pode não ser suficiente para gerar uma conclusão, que requer uma série de casos. No entanto, este caso destaca um método inovador que pode ser praticado de forma eficaz e econômica. Isso estabelece um precedente para futuros avanços na oncologia ortopédica em todo o mundo, especialmente em países com restrições financeiras em relação à assistência médica.
